# Fast reprogramming and adaptive reproduction of contact-rich assembly

**DOI:** 10.3389/frobt.2026.1746577

**Published:** 2026-03-18

**Authors:** Dimitrios Rakovitis, Vamsi Krishna Origanti, Vinzenz Bargsten, Adrian Danzglock, Dennis Mronga, Frank Kirchner

**Affiliations:** 1 German Research Center for Artificial Intelligence (DFKI GmbH), Robotics Innovation Center, Bremen, Germany; 2 Department of Mathematics and Informatics, University of Bremen, Bremen, Germany

**Keywords:** adaptive model predictive control, adaptive resonance theory, agile manufacturing, contact-rich assembly, dynamic movement primitives

## Abstract

**Introduction:**

Modern manufacturing demands flexible, robust robotic assembly systems capable of handling variable part geometries and dynamic task configurations. Current approaches often suffer from limited generalization, high sample complexity, and the need for extensive reconfiguration or retraining when task parameters change. This paper addresses these limitations by introducing a novel framework that enables adaptive reproduction of kinesthetically taught, contact-rich assembly policies, using only force/torque and proprioceptive sensing.

**Methods:**

The approach combines three components: i. synchronized wrench–motion Dynamic Movement Primitives (wDMPs) that encode coupled motion and wrench profiles from a single demonstration; ii. an uncertainty-aware Model Predictive Controller (MPC) that updates its model online to enable compliant and adaptive contact handling using uncertainty estimated via a Gaussian Mixture Model (GMM); and iii. a neural contact classifier based on Adaptive Resonance Theory (ART) that distinguishes intended contacts from unintended misalignments and coordinates transitions between assembly stages.

**Results and Discussion:**

Trained on just two demonstrations, one kinesthetic teaching and one assisted successful reproduction, the framework was evaluated on standard benchmarks and real-world industrial scenarios, including peg-in-hole, plug insertion, and disc brake assemblies. Across 47 assemblies, our framework increased the success rate from 29.8% to 83% in comparison to a classic, nonadaptive compliant controller, and demonstrated improved robustness and transferability over baseline controllers under geometric and pose variations. This contributes towards enabling agile, customizable production with minimal reprogramming effort.

## 1 Introduction

Modern manufacturing increasingly demands the ability to produce a diverse range of products, requiring assembly systems to handle frequent changes in part geometry, fixture configurations, and sequencing. This variability challenges traditional robotic assembly solutions, which are typically finely engineered or trained for well-defined tasks and variations (Luo et al., 2019; Tang et al., 2023; Noseworthy et al., 2025; Guo et al., 2025; Wu et al., 2025; Yan et al., 2021; Jha et al., 2022; Schoettler et al., 2020; Goyal et al., 2024; Kim et al., 2020; Chang et al., 2022; Morgan et al., 2023). Even then, such solutions are often labor-intensive and time-consuming, which underscores the need for systems capable of robust, adaptive behavior with minimal manual reprogramming.

To address these challenges, we propose a novel framework that enables fast, intuitive robot programming and adaptive reproduction of previously unseen, contact-rich assembly tasks using only a force/torque (F/T) sensor and error measurements. The method integrates Dynamic Movement Primitives (DMPs), Adaptive Model predictive Control (AMPC), and an Adaptive Resonance Theory (ART)–based contact classifier. DMPs encode human-taught assembly demonstrations as stable nonlinear dynamical systems, which capture the motions and wrenches required to perform a task and enable smooth generalization to new initial states and goal fixture configurations (Ijspeert et al., 2013; Kramberger et al., 2017; Steinmetz et al., 2015). AMPC is a model-based predictive controller (MPC) whose prediction model is updated online from data (Rakovitis and Mronga, 2024), enabling adaptive and compliant resolution of errors, such as misalignments caused by unexpected contacts during reproduction of the learned assembly. An ART-based contact classifier is a neural network (NN) that incrementally classifies F/T patterns under a vigilance criterion to recognize known contacts and flag novel ones on the fly (Bargsten et al., 2025). These three methods are combined as follows.

For each new assembly task family (e.g., peg-in-hole or plug-insertion), the approach lets a user train the system in two demonstrations (fast reprogramming): i. a kinesthetic teaching, and ii. an assistive reproduction. At first, the required coupled motion and wrench profiles are captured from a single nominal demonstration using synchronized wrench-motion DMPs (wDMPs). The user segments the demo into the necessary sequences, so that a separate wDMP is trained for i. aligning the parts with the fixture and ii. inserting the parts. Then, each wDMP provides MPC with the motion and wrench references needed to reproduce the learned task. In the second step, an assistive reproduction is executed for the same goal pose and parts, using MPC which models the interaction dynamics with a Cartesian Impedance Model (CIM). During this demo, the robot collects contact data (error-F/T measurements) from a human-ensured, successful assembly, for training ART to recognize correctly aligned contact patterns, and for fitting a Gaussian Mixture Model (GMM) for uncertainty estimation under nominal operation. This GMM later provides uncertainty estimates for new, autonomous assembly executions, by evaluating the likelihood of incoming observations w.r.t. a successful assembly. We refer to this training procedure as “fast reprogramming”, because each task needs to be taught only once and reproduced successfully once, reducing the required training time to essentially the length of the two demonstrations.

**FIGURE 1:**
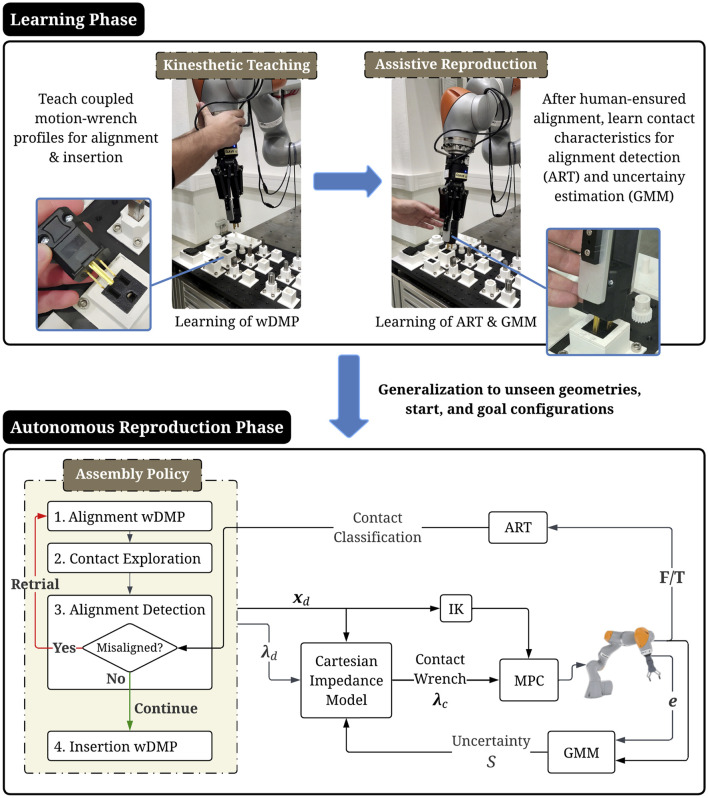
Graphical abstract of the proposed framework.

After learning, the method is deployed on a real system to autonomously execute diverse, previously unseen assembly tasks with varying geometries, start and goal poses (autonomous reproduction phase). In this setting, misalignments and unintened contacts caused by modelling or goal-estimation errors are inevitable. To address this, after an alignment attempt and before the actual part insertion, the contact surface is explored by applying sinusoidal dither forces at the EE, about the estimated goal direction, guided by tracking errors. The ART classifier monitors F/T data to flag undesired contacts and acts as a scheduler, triggering insertion upon confident alignment or initiating a retrial when the context deviates from known patterns. Throughout, the estimated GMM uncertainty adapts the MPC model based on the distribution of error-F/T measurements, enabling compliant and adaptive exploratory behavior in case of misalignments. A graphical abstract of the proposed approach can be seen in Figure 1.

Despite recent progress in robotic assembly, most of the literature focuses on learning or engineering nominal policies, while the problem of reliably reproducing such policies under variations in parts and real-world uncertainties, such as modeling errors and localization inaccuracies, remains comparatively underexplored. To the best of our knowledge, our method is the first to combine fast programming from only two demonstrations, uncertainty-aware adaptive compliance within MPC, and online contact-context classification that regulates stage transitions and triggers retrials. Together, these components are designed to reduce reprogramming effort, while improving the chances of successful reproduction of the learned policy in the context of real world, contact-rich assembly under variations.

The approach is evaluated experimentally using a 7-DOF KUKA LBR iiwa14 R820 manipulator, equipped with a F/T sensor at the wrist. The robot performs a wide range of contact-rich assembly tasks, starting with the standard IndustRealKit benchmark (Tang et al., 2023). This includes classic peg-in-hole tasks with cylindrical and rectangular pegs of varying sizes, two- and three-prong plug insertions (which require significant insertion force), and multi-stage gear assemblies involving both peg insertion and gear teeth alignment. Beyond these benchmarks, we evaluate the system on a real-world industrial scenario: a multi-stage disc brake assembly, requiring the handling of heavy components and the application of substantial forces. Each task is repeated across multiple trials to obtain statistically meaningful results. As our novelty lies primarly in the adaptive reproduction of kinesthetically taught policies, we benchmark our reproduction scheme against two state-of-the-art baseline controllers (classic Cartesian impedance control (Origanti et al., 2025), and error-based predictive variable impedance control (Anand et al., 2023)), as well as ablation baselines to highlight the importance of key components during reproduction.

Overall, the contributions of this work are listed below:

- We propose a novel, user-friendly framework for fast programming and adaptive reproduction of contact-rich robotic assembly tasks, that combines wDMPs with AMPC. This combination allows robust generalization to varying start and goal assembly configurations via wDMPs, as well as to different part geometries via AMPC, which enables compliant and adaptive contact handling based on uncertainty.
- We combine the above with an ART-based neural contact classifier for real-time detection of undesired contact patterns and misalignments, which also serves as a scheduler for assembly stage transitions and retrials.
- We provide comprehensive experimental validation on diverse industrial and benchmark scenarios, demonstrating improved handling of misalignments, and adaptability over baseline approaches.

The remainder of the paper is structured as follows: Section 2 details related works, Section 3 outlines the proposed methodology, Section 4 discusses the experimental results, and Section 5 concludes with a summary of findings and potential directions for future work.

## 2 Related work

Prior research on learning, controls, and contact classification for contact-rich tasks is detailed in the following subsections.

### 2.1 Related works on learning for assembly

Recent research in robotic assembly has explored a wide range of learning-based approaches to address the challenges of contact-rich insertion tasks. Reinforcement learning (RL) methods have shown promise for learning robust, low-tolerance behaviors, e.g., with Guided policy search (Levine et al., 2015), and RL-based variable impedance control (Luo et al., 2019). Simulation-based RL pipelines such as IndustReal (Tang et al., 2023) and FORGE (Noseworthy et al., 2025) have demonstrated successful sim-to-real transfer with tight-tolerance insertions, through domain randomization, signed-distance rewards, or force conditioning. Meta-RL approaches (Schoettler et al., 2020) leverage shared structure across insertion tasks to enable rapid adaptation from simulation to real-world scenarios. Similarly, SRSA (Guo et al., 2025) retrieves relevant skills from a pre-existing policy library using predicted transfer success, and fine-tunes them on new assembly tasks using Proximal Policy Optimization (PPO) combined with self-imitation learning.

Supervised and imitation learning approaches have also proven effective. In Yan et al. (2021), a contact-state recognition model is trained on F/T data, collected from various inclined peg-in-hole configurations. A support vector machine (SVM) with a Gaussian kernel enables accurate classification of the contact state, which then informs the parameters of an adaptive impedance controller to compliantly correct misalignments. In Jha et al. (2022), DMPs are used to learn a nominal insertion trajectory from human demonstrations, with a corrective compliance policy to handle vision-based goal estimation errors. A generalized accommodation controller bounds contact forces for safe exploration and data collection, while a Gaussian Process (GP) trained on F/T data predicts misalignments to guide successful insertions despite pose errors. DMPs (Ijspeert et al., 2013) have been extensively adapted to represent forces and complex, multi-modal behaviors. Several studies have incorporated force feedback into DMP frameworks (Pastor et al., 2009; Hoffmann et al., 2009), while others have employed basis function regression to model and reproduce force profiles (Ude et al., 2010; Kramberger et al., 2017). Extensions such as Task-Parameterized DMPs (Calinon, 2016) and Probabilistic Movement Primitives (ProMPs) (Paraschos et al., 2013) enhance spatial generalization and capture variability across several demonstrations. Origanti et al. (2022) showcases automatic extraction of skill parameters from human demonstrations, to replicate both motion and force trajectories in simulation, particularly for contact-rich manipulation. Complementarily, RVT-2 (Goyal et al., 2024) introduces a multi-view vision-based system that learns high-precision manipulation from just a few demonstrations. It uses supervised behavioral cloning to map a third-person RGB-D input and language instructions to key-frame poses, with a multi-view transformer and a coarse-to-fine inference strategy enabling millimeter-level accuracy using only visual data. TacDiffusion (Wu et al., 2025) leverages diffusion models trained on expert demonstrations using cuboid pegs to map tactile observations to force-domain actions, achieving a 95.7% zero-shot transfer success rate on novel peg-in-hole tasks including cylinder, prism, and key-shaped pegs.

Imitation learning has also been combined with RL in Kim et al. (2020) by using a human demonstration to train a NN-based movement primitive (NNMP), which constrains RL to a known trajectory manifold. With a properly designed reward function, this enabled the agent to learn high-precision contact tasks while minimizing applied forces. In Chang et al. (2022), an assembly task learned via motion-force DMPs is reproduced with a low-level admittance controller, whose stiffness is tuned by RL, enabling real-time impedance adaptation. Other works, using compliance-enabled strategies (Morgan et al., 2023) or neglecting force feedback (Park et al., 2017) offer hardware-efficient alternatives that exploit passive compliance or implicit search behaviors.

Despite these advancements, several limitations are shared across the above works. Many approaches are sample-inefficient, requiring extensive manual training or simulations that can take several hours to days (Luo et al., 2019; Tang et al., 2023; Noseworthy et al., 2025; Guo et al., 2025; Wu et al., 2025; Yan et al., 2021; Jha et al., 2022; Calinon, 2016; Paraschos et al., 2013; Kramberger et al., 2017), or additional multiple real-world trials (≈10−30) for transfer learning (Schoettler et al., 2020; Goyal et al., 2024; Kim et al., 2020; Chang et al., 2022). Generalization to arbitrary geometries or unseen part configurations is limited, often requiring timely retraining, fine re-engineering (Morgan et al., 2023), or even specifically engineered sensing conditions such as calibrated multi-view camera rigs (Goyal et al., 2024). Additionally, many of the approaches, consider cut-off forces or fixed compliance (Schoettler et al., 2020; Park et al., 2017; Morgan et al., 2023; Noseworthy et al., 2025) for safe exploration, which may result in task failure in diverse tasks involving significantly different interaction forces, e.g., resistant plug insertion or sliding on high friction surfaces. Adapting these systems for rapidly changing contact-rich assembly tasks, typically entails significant reconfiguration. For example, in a manufacturing environment this would result in costly extended production downtime (Liu et al., 2012), whenever small changes in production are introduced. This highlights the need for flexible, intuitive, and data-efficient methods that can rapidly adapt to diverse changes in assembly settings. For these reasons, in this work we draw inspiration from Steinmetz et al. (2015) and extend standard DMPs (Fabisch, 2024) to learn coupled motion–wrench profiles from a single Cartesian space demonstration. We then reproduce these profiles with an adaptive controller, enhancing generalization to varying part geometries, start and goal configurations.

### 2.2 Related works on controls

Typically the above works rely on compliant systems (passive or active), with many of them using Cartesian Impedance Control (CIC) to introduce the required task space compliance for safe contact interaction. However, CIC is inherently reactive; it lacks prediction and cannot natively enforce constraints (e.g., joint/torque limits), so it often relies on heuristic task-specific safety margins (Origanti et al., 2025).

Recent (non-assembly) studies combine CIC with MPC into model-based impedance- (MPIC) (Bednarczyk et al., 2020) or variable-impedance control (MPVIC) (Thelenberg and Ott, 2024; Anand et al., 2023) for tasks involving contact uncertainties or variable stiffness objectives. By embedding impedance dynamics in MPC, these approaches retain compliance while enabling look-ahead planning, explicit constraint handling, and proactive adaptation across contact transitions. In particular Thelenberg and Ott (2024) and Anand et al. (2023), leverage the impedance model inside the MPC to forecast the stiffness and damping commanded by a low-level variable-impedance controller, improving task adaptability.

However Bednarczyk et al. (2020), enforces a fixed stiffness profile over the entire task, limiting the controller’s ability to adapt compliance dynamically based on environmental conditions or task phases. Conversely Thelenberg and Ott (2024) and Anand et al. (2023), drive stiffness adaptation by the magnitude of tracking errors, increasing stiffness when the robot is far from its target. While intuitive, this heuristic can lead to unphysical or unsafe stiffness changes in contact-rich scenarios, where increased stiffness at the wrong moment may cause damage, or task failure during contact transitions.

A related line of work addresses contact uncertainty using adaptive MPC, in which the prediction model is updated online based on an estimated contact model. This contact model is either learned, e.g., via RL (Xu S. et al., 2022), supervised learning (Rakovitis and Mronga, 2024), or meta-learning (Saviolo et al., 2024; Anne et al., 2021; Arcari et al., 2023), or predicted via adaptive control or system-identification techniques (Minniti et al., 2021; Xu J. et al., 2022). Such approaches have mainly been demonstrated on mobile manipulators, quadrupeds, and quadrotors, where they are used to compensate for unknown or time-varying dynamics, including payload changes and external disturbances.

Although, all the above control approaches are achieving great results on dealing with contact uncertainties, their effectiveness on fine assembly tasks have yet to be validated, where high precision, delicate contact handling, and sub-millimeter accuracy are required. This highlights the need for more robust studies on context-aware controllers that account for both contact dynamics and compliance requirements to succeed across fine and diverse assembly tasks. Hence, in this work we extend MPC with a Cartesian impedance contact model, whose stiffness and desired wrench are continuously adapted based on uncertainty estimates. These uncertainties are derived from the likelihood of the observed errors and wrench measurements under a GMM trained on a nominal (successful) execution. This measures the proximity of the current situation to an out-of-distribution (OOD) case, thereby enabling adaptive and compliant reproduction of the learned assembly task when unexpected interactions occur. We employ GMMs, as they have been shown to be highly sample-efficient, especially in high-dimensional spaces, and very fast to train (Calinon et al., 2007; Rakovitis and Mronga, 2024). GMMs have also been used before for anomaly and OOD detection (Zong et al., 2018; Iwata and Kumagai, 2022).

### 2.3 Related works on contact classification

The detection and classification of contacts between a robot and its environment is essential for inferring the current state of the system. Prior research broadly falls into two focal points. The first addresses the detection of unintended collisions, aiming to mitigate impact forces via reflexive counter-actions. Classically, this type of approaches compare an estimated impact metric against a pre-defined threshold. Because robotic assembly inherently involves purposeful contact, our work aligns with the second focal point: the classification of intended contacts to obtain a more fine-grained assessment of the contact forces when performing an assembly task.

Several works have tried to bridge the gap between the two focal points by jointly distinguishing unintended collisions from intended interactions. For example (Cho et al., 2012), differentiates collisions from intended contact by monitoring the rate of change in joint torque measurements, while frequency-domain analyses have also shown promise (Kouris et al., 2016; 2018). In robotic assembly, however, the classification into only two classes (three with no-contact class) based on thresholds is insufficient to assess the successful insertion and joining of rigid and elastic parts with tight tolerances. To address this, some works such as Pankert and Hutter (2023) incorporate visual monitoring with prior knowledge to improve kinematic-level performance by fusing CAD models, tactile cues, and particle simulation to refine object localization.

In contrast, our objective is an assessment driven by contact wrenches, that does not depend on precise prior models or expert supervision. Other domains underscore the potential of such signals, including classification from joint torque measurements (Iskandar et al., 2024) and from acoustic vibration sensing (Liu and Chen, 2024). Within robotic assembly specifically, two preliminary works have shown the feasibility of employing an incremental machine learning approach for continuous classification of episodes encoded by the frequency magnitudes of joint torque measurements (Bargsten and Kirchner, 2023) or EE wrench measurements (Bargsten et al., 2025). In these approaches, time-series measurements are encoded using a short-time Fourier transform (STFT), producing compact, episodic signatures that lend themselves to real-time classification via Adaptive Resonance Theory (ART).

ART originates in cognitive science and models dynamic processes for learning and adapting short- and long-term memory in the human brain (Grossberg, 1976; Carpenter and Grossberg, 1987). ART’s learning principle is a match-based learning that relies on input similarity, in contrast to error-based batch learning such as backpropagation. Therefore, ART naturally supports continuous learning of novel patterns without catastrophic forgetting, and allows to capture rare input events. Building on this foundation, numerous algorithmic variants (Brito da Silva et al., 2019) have been developed that simplify and operationalize these principles. To this end, we adopt Distributed Dual Vigilance Fuzzy ART (DDVFA) (Brito da Silva et al., 2020), a variant of ART in which each learned class is represented by a Fuzzy ART (Carpenter et al., 1991) NN, i.e., an unsupervised clustering model that measures input similarity via fuzzy set operations. This nested design thus represents classes as groups of sub-classes and is able to capture arbitrarily shaped, heterogeneous clusters in the data. Differently from the prior applications, we leverage this property to use DDVFA as a context monitor, classifying contact patterns during assembly and triggering transitions between assembly stages.

## 3 Methodology

This section describes the proposed methodology in five subsections covering: 1. the DMPs formulation, 2. a force-based contact exploration strategy, 3. the contact classification module via ART, 4. the MPC problem, and 5. the estimation of uncertainty via a GMM and its use to adapt MPC in real-time. Our framework consists of two parts: i. the learning, and ii. the reproduction phase. The complete pipelines for each phase, are detailed in Algorithm 1 and Algorithm 2, respectively.

Algorithm 1 **Learning of contact-rich assembly.**

**Input** **:** Single kinesthetic demonstration $D={\left\{\left(x^{\left(i\right)},\eta^{\left(i\right)},\lambda^{\left(i\right)}\right)\right\}}_{i=1}^{N}$ in gravity mode
**Output:** wDMPs, ART classifier, GMM
Learning is performed in 2 demos:
**1) Kinesthetic teaching**
  **1.1)** Learn forcing terms $\left\{f_{\text{x}},f_{\eta },f_{\lambda }\right\}$ (Equation 3) of DMPs (Equations 1–9) from D .
  **1.2)** Segment demo into *alignment* and *insertion* → fit one wDMP per segment.
**2) Assistive reproduction** (with MPC (Section 3.4))
  **2.1)** *Execute alignment wDMP,* with human assistance:
   – wDMPs → desired pose and wrench → MPC.
   – Human → minimal corrections → ensure parts alignment.
  **2.2)** *Once aligned* → axial dither on assembly Z-axis (Equation 12) → collect contact F/T data → train ART classifier (Section 3.3).
  **2.3)** *Execute insertion wDMP*: collect error–wrench data under nominal insertion in $D={\left\{z^{\left(i\right)}=\left[e^{\left(i\right)},\lambda_{\text{m}}^{\left(i\right)}\right]\right\}}_{i=1}^{N}\;\to$ fit a GMM (Equation 19) on D .

Algorithm 2 Adaptive autonomous reproduction of contact-rich assembly.

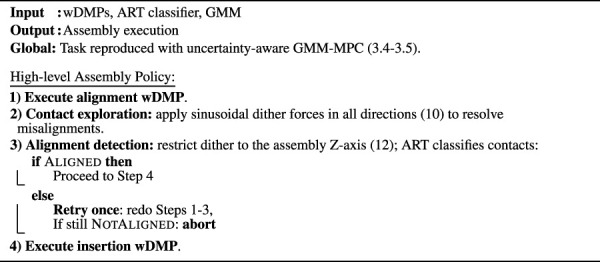

### 3.1 Dynamic movement primitives

Each assembly task is specified by the parts to be assembled (whose geometry and required interaction forces may be unknown), along with an initial pose and a fixed goal pose in Cartesian space (which are known). Hence, each task is encoded by two DMPs that share a single phase variable, thereby synchronizing pose and wrench generation over time:

1. a Cartesian DMP that generates EE pose trajectories (3D position and 4D quaternion),
2. a Wrench DMP that reproduces the EE wrench (3D force and 3D torque).

We refer to this pair as a *synchronized wrench–motion DMP* (*wDMP*). By jointly learning motion and the associated interaction wrenches, wDMPs extend standard DMPs (Ijspeert et al., 2013; Fabisch, 2024) from pure kinematics to coupled motion-wrench behavior, enabling a robot to acquire the spatial and wrench dynamics required for contact-rich manipulation from a single demonstration.

#### 3.1.1 Cartesian DMP

We follow the formulation in Fabisch (2024), which supports Cartesian trajectories, position $x\in R^{3}$ and orientation as quaternions η∈H,‖η‖=1 , to ensure smooth interpolation on the unit sphere.

The position evolves according to the standard second-order dynamical system:

$$\tau \dot{x}=v_{\text{x}}$$ (1)

$$\tau {\dot{v}}_{\text{x}}=\alpha_{\text{x}}\left(\beta_{\text{x}}\left(g_{\text{x}}-x\right)-v_{\text{x}}\right)+f_{\text{x}}\left(s\right)$$ (2)

where $v_{\text{x}}$ is an auxiliary state representing the Cartesian velocity, $g_{\text{x}}$ is the goal position, τ is the temporal scaling factor, $\alpha_{\text{x}}$ and $\beta_{\text{x}}$ are positive gains, and $f_{\text{x}}\left(s\right)$ is the nonlinear forcing term learned from demonstration and represented using a weighted sum of N radial basis functions (RBFs). A forcing term is given by:

$$f\left(s\right)=\frac{\sum_{i=1}^{N}\psi_{i}\left(s\right)\;w_{i}}{\sum_{i=1}^{N}\psi_{i}\left(s\right)},\;\psi_{i}\left(s\right)=\exp\left(-h_{i}{\left(s-c_{i}\right)}^{2}\right),$$ (3)

where $w_{i}$ are the learned weights, $c_{i}$ are the centers, and $h_{i}$ are widths of the basis functions. The phase variable s evolves over time according to the canonical system:

$$\tau \dot{s}=-\alpha_{s}s,$$ (4)

with $\alpha_{s}>0$ .

For orientation, using the full quaternion error formulation (Ude et al., 2014; Fabisch, 2024), we write.

$$\tau \dot{\omega }=\alpha_{\omega }\;\delta \left(\eta ,\eta_{d}\right)-\beta_{\omega }\;\omega -\alpha_{\omega }\;\delta \left(\eta_{0},\eta_{d}\right)\;s+\alpha_{\omega }\;f_{\eta }\left(s\right),$$ (5)

$$\tau \dot{\eta }=\frac{1}{2}\;\omega ⊗\eta .$$ (6)

Here, $\omega \in R^{3}$ is the angular velocity, $\eta_{0}$ and $\eta_{d}$ denote respectively the initial and desired (goal) quaternions, and $f_{\eta }$ is the respective forcing term. The positive constants $\alpha_{\omega }$ and $\beta_{\omega }$ control the convergence rate and damping, respectively. The logarithmic quaternion error is

$$\delta \left(\eta_{1},\eta_{2}\right)=2\;\log\;\left(\eta_{2}⊗{\bar{\eta }}_{1}\right),$$ (7)

with ⊗ the quaternion product, ${\bar{\eta }}_{1}$ the conjugate of $\eta_{1}$ , and log(⋅) the $S^{3}\;\to \;so\left(3\right)$ logarithmic map. This formulation constrains the trajectory to the unit quaternion manifold, avoiding singularities and enabling consistent learning and reproduction.

#### 3.1.2 Wrench DMP

To model contact interaction, a similar DMP is used for the wrench vector $\lambda \in R^{6}$ :

$$\tau \dot{\lambda }=v_{\lambda }$$ (8)

$$\tau {\dot{v}}_{\lambda }=\alpha_{\lambda }\left(\beta_{\lambda }\left(g_{\lambda }-\lambda \right)-v_{\lambda }\right)+f_{\lambda }\left(s\right),$$ (9)

where $g_{\lambda }$ is the goal wrench, the forcing term $f_{\lambda }$ is learned componentwise for all six dimensions, and all other parameters are similarly defined as in Cartesian DMP. This DMP shares the same phase variable s as the Cartesian DMP to ensure time alignment between motion and wrench evolution (Gams et al., 2014).

#### 3.1.3 Training of wDMPs

For each novel assembly, a human performs kinesthetic teaching of the robot in gravity-compensation mode to provide a single demonstration. The measured pose and wrench trajectories are used to fit the forcing terms $f_{\text{x}}$ , $f_{\eta }$ , $f_{\lambda }$ (Equation 3) via imitation learning using ridge regression (Hoerl and Kennard, 1970). Assuming that each assembly task consists of two phases: i. alignment with the fixture, and ii. part insertion, the user segments the demonstration so that one wDMP is obtained for each phase (Equations 1–9). At execution time, the Cartesian DMP yields the desired EE pose trajectory, while the Wrench DMP simultaneously produces the expected interaction wrench.

### 3.2 Contact exploration via force-driven dither motions

Having an expert-learned wDMP is not enough to guarantee a successful reproduction of the learned assembly. In reality, small misalignments during reproduction could occur due to modeling inaccuracies or minor geometric mismatches. To overcome this issue, an error-driven, force-based exploration policy is employed between the alignment and insertion phase. To facilitate local contact exploration, a time-varying dither force $F_{c,exp}\left(t\right)\in R^{3}$ is applied at the EE. This force consists of sinusoidal components along each Cartesian direction:

$$\begin{array}{cc} F_{c,exp}^{\left(i\right)}\left(t\right) & =A_{i}\sin\left(2\pi f_{i}t\right)\cdot \left(d_{\text{nom,i}}+\xi \cdot \text{sign}\left(d_{\text{nom,i}}\right)\right),\\ i & \in \left\{x,y,z\right\} \end{array}$$ (10)

where $A_{i}$ and $f_{i}$ are the amplitude and frequency of the sinusoidal force in the i -th direction, and 0<ξ≪1 is a small perturbation term added to prevent the exploration force from vanishing near the goal. The probing direction $d_{\text{nom}}$ is computed as the normalized vector:

$$d_{\text{nom}}=\frac{p_{g}-r_{EE}}{\left\|p_{g}-r_{EE}\right\|}.$$ (11)

In Equation 11 $p_{g}$ denotes the estimated goal position (e.g., hole center), and $r_{EE}$ is the current EE position obtained from forward kinematics. Selecting a low-frequency for these forces allows exploration of the contact surface, while a high-frequency refines an existing contact. These dither motions generate interaction forces that assist in resolving edge contacts and overcoming tight clearances.

Consequently, an alignment detection step is required to check if the exploration strategy resolved the misalignment after a fixed number of attempts or time interval. For this purpose, a similar dither force $F_{c,ad}\left(t\right)\in R^{3}$ is applied, but only in the assembly direction ${\hat{n}}_{d}$ given by wDMP. This is:

$$\begin{array}{cc} F_{c,ad}\left(t\right) & =\left(F_{c,ad}^{\;\min}+\frac{F_{c,ad}^{\;\max}-F_{c,ad}^{\;\min}}{1+exp\left(\alpha_{F}\left(c_{F}-sin_{F}\right)\right)}\right){\hat{n}}_{d},\\ sin_{F} & =\frac{\sin\left(2\pi \;f_{F}\;t\right)+1}{2} \end{array}$$ (12)

where $F_{c,ad}^{\;\min},\;F_{c,ad}^{\;\max}\leq 0$ are the bounds of the applied force, $\alpha_{F}<0,\;c_{F}\in \left[0,1\right]$ are logistic parameters, and $f_{F}$ is its frequency. These parameters, along with the ones in Equation 10, must be tuned empirically according to the desired outcome for the given robotic system.

To compute the full desired contact wrench for contact exploration, the corresponding contact torques are given as, $\tau_{c}=r_{EE}\times F_{c}\in R^{3}$ . The exploration process continues until a maximum number of attempts is reached or a successful alignment is detected by the ART-based contact classifier described next.

### 3.3 Contact classification via distributed dual vigilance fuzzy ART (DDVFA)

The role of the ART classifier in our framework is to verify that the parts to be assembled are properly aligned before joining them, in order to prevent jamming caused by insertion forces applied at incorrect locations. In an ART network, learning proceeds in cycles, governed by an *orienting subsystem,* which controls the activation and inhibition of nodes representing the categories (classes) during a *competition* for the best match to the input. Besides the orienting subsystem, a typical ART NN consists of fields (layers) $F_{1}$ and $F_{2}$ that are connected through weight vectors, as well as an optional $F_{0}$ field. A cycle can then be described as follows:

1. **Input encoding.** The raw input vector is optionally preprocessed or encoded in the $F_{0}$ field before being presented to the feature representation field $F_{1}$ .
2. **Category activation.** This vector is then forwarded through bottom-up weights from the $F_{1}$ field to $F_{2}$ , the category representation field. Each *node* in $F_{2}$ is a computational unit (neuron) representing a learned category (class) via its weight vectors.
3. **Competition and choice.** The $F_{2}$ nodes compete for the highest activation for the current input. The best candidate is selected and the other nodes are inhibited.
4. **Resonance (similarity) test.** The chosen $F_{2}$ node sends a top-down expectation pattern back to $F_{1}$ through its top-down weights. The orienting subsystem compares this expectation with the actual input in $F_{1}$ . If their similarity exceeds a vigilance threshold, the node enters a resonant state.
5. **Learning.** When the similarity test is satisfied (resonance), the selected node’s bottom-up and top-down weights are updated, thereby incorporating the input into long-term memory. Otherwise, the orienting subsystem suppresses this node and continues the search with the node having the next-highest activation. If none of the nodes achieves a resonant state, a new $F_{2}$ node is created to represent a novel category in memory.

The DDVFA variant of ART implements a nested approach, in which the classes are represented by nodes that are themselves FuzzyART NNs. In FuzzyART, each input feature vector $\chi \in R^{n},\;0\leq \chi_{i}\leq 1\;\forall \;i$ is firstly *complement coded* when passing through the $F_{0}$ field, i.e., $I={\left(\chi ,1-\chi \right)}^{⊤}$ . Using this encoded feature vector, each node j then determines its activation $T_{j}$ based on its weight vector $w_{j}$ which serves as the memory:

$$T_{j}=\frac{\|I\wedge w_{j}{\|}_{1}}{\alpha_{T}+\|w_{j}{\|}_{1}},$$ (13)

where $\|\cdot {\|}_{1}$ is the $L_{1}$ norm, ∧ denotes the fuzzy set AND operator (intersection, element-wise minimum), and $\alpha_{T}$ biases selection toward uncommitted nodes.

Nodes are considered in descending order of $T_{j}$ until one satisfies the resonance criterion

$$M_{j}=\frac{\|I\wedge w_{j}{\|}_{1}}{\|I{\|}_{1}}\geq \rho ,$$ (14)

with vigilance parameter ρ∈[0,1] and match value $M_{j}$ (similarity). The node j that passes the resonance test, updates its weights according to

$$w_{j}^{\text{new}}=\left(1-b\right)\;w_{j}^{\text{old}}+b\;\left(I\wedge w_{j}^{\text{old}}\right),$$ (15)

with learning rate b∈[0,1] . In DDVFA, a secondary, less strict global vigilance parameter extends this mechanism to regulate learning at the level of grouped classes.

**FIGURE 2:**
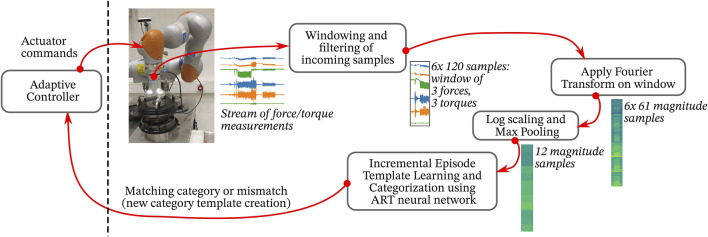
Dataflow of the ART-based contact learning and classification.

In this work, DDVFA learns and classifies data from a stream of EE wrench samples composed of force and torque vectors, each $\in R^{3}$ . Before entering the ART network, the wrench stream is preprocessed as follows: i. consecutive sample blocks are windowed with a Blackman–Harris window and transformed to the frequency domain via the Fast Fourier Transform (FFT); ii. the number of features is reduced by max-pooling on the resulting frequency magnitudes; and iii. all features are scaled to [0,1]. The resulting feature vectors are then presented to the ART NN. The overall pipeline is illustrated in Figure 2.

In our framework, ART is trained during the assistive reproduction, after a wDMP has been learned. During the alignment phase, the human guides the robot slightly to align properly in case of millimeter errors. After correct alignment is ensured, Equation 12 is applied to collect F/T data characterizing correct alignment with the assembly fixture. These data are used as input to the learning and classification procedure outlined above (Equations 13–15). This results in learning a set of template classes which map to episodes in the contacts of the assembly process. When the learning and adaption is further only activated during the successful alignment check, a distinct learning and further recognition of the frequency magnitude pattern of successful alignment is achieved, mapping all other patterns to a mismatch (category index −1 ). The trained classifier is then frozen and deployed as a real-time context monitor and scheduler, triggering insertion after identifying correct alignment or a retrial otherwise, enabling adaptive stage transitions during contact-rich assembly.

### 3.4 Model predictive control

In this work, MPC is used to reproduce the assembly task. For a manipulator with n joints, the MPC problem is expressed in joint-space as in (Rakovitis and Mronga, 2024):

$$\begin{array}{cc} \underset{x,u}{\text{min}}\; & \|x_{d}\left(T\right)-x\left(T\right){\|}_{Q_{T}}^{2}+\int_{0}^{T}\left(\|x_{d}-x{\|}_{Q}^{2}+\|u{\|}_{R}^{2}\right)dt\\ \text{s.t.} & \dot{x}=f\left(x,u\right)\\ \; & x\left(0\right)=x_{0}\\ \; & x_{\min}\leq x\leq x_{\max}\\ \; & u_{\min}\leq u\leq u_{\max} \end{array}$$ (16)

where, the system state is denoted by $x=\left(q,\dot{q}\right)$ , encompassing joint positions and velocities; u=τ represents the control input in the form of joint torques; $x_{d}$ denotes the joint-space reference obtained via Inverse Kinematics (IK) on the state-space reference given by the Cartesian DMP; $x_{0}$ is the initial state; and T represents the prediction horizon. The constraints ensure that both the state and input remain within defined bounds $x_{\min}$ , $x_{\max}$ , $u_{\min}$ , $u_{\max}$ . Assuming a rigid contact at the EE, the dynamics f are given by:

$$H\left(q\right)\;\ddot{q}+C\left(q,\dot{q}\right)=\tau +J_{c}^{⊤}\lambda_{c}.$$ (17)

here, H(q) is the inertia matrix in joint space, $\ddot{q}$ is the joint acceleration, $C\left(q,\dot{q}\right)$ represents the Coriolis, centrifugal, and gravitational effects, and $J_{c}$ is the Jacobian at the contact point. The contact wrench $\lambda_{c}\in R^{6}$ is modeled using a CIM:

$$\lambda_{c}=Ke_{\text{x}}+D{\dot{e}}_{\text{x}}+\lambda_{d},$$ (18)

where the stiffness and damping K , $D\;\in R^{6\times 6}$ , are diagonal, positive semi-definite matrices. Those control the state-space compliance w.r.t. the EE pose and twist errors $e_{\text{x}}$ , ${\dot{e}}_{\text{x}}$ . The wrench $\lambda_{d}$ is the desired wrench applied at the EE, provided in real-time either by the Wrench DMP or by the force-based contact exploration policy, according to the stage of the assembly. This formulation is chosen as it allows to control the compliance in state-space by adjusting $\lambda_{c}$ , while preserving postural joint-space control. Moreover, to simplify the adaptation of compliance in the system we set $D=2\zeta \sqrt{K}$ , with 0<ζ<1 . The contact wrench $\lambda_{c}$ is adapted at each time-step before solving MPC (Equations 16, 17) based on operation uncertainty, enabling compliant and adaptive handling of unexpected contacts as outlined in the next subsection.

### 3.5 Uncertainty estimation

The uncertainty during execution of an assembly operation is derived using a GMM, trained during the human-assisted reproduction. After correct alignment is ensured by the operator and data collection for ART has been completed, the insertion phase takes place. The observed Cartesian pose errors and wrenches recorded at the EE during this phase are collected into a dataset $D={\left\{z^{\left(i\right)}=\left[e^{\left(i\right)},\lambda_{\text{m}}^{\left(i\right)}\right]\right\}}_{i=1}^{N}$ , where $e=\left[e_{\text{x}}^{⊤},{\dot{e}}_{\text{x}}^{⊤}\right]$ denotes the error measurements, and $\lambda_{\text{m}}=\left[F_{\text{m}}^{⊤},\tau_{\text{m}}^{⊤}\right]$ represents the F/T sensor measurements. This dataset is used to train a GMM with Expectation Maximization (Dempster et al., 1977), to model the joint distribution of the error-F/T measurements. A GMM is defined as

$$p\left(z\right)=\overset{K}{\underset{k=1}{\sum }}\pi_{k}\;N_{k}\left(z\;|\;\mu_{z}^{k},\Sigma_{z}^{k}\right)$$ (19)

where $N_{k}$ denotes a multivariate Gaussian distribution with mean $\mu_{z}^{k}$ and covariance matrix $\Sigma_{z}^{k}$ . Each component is weighted by a prior $\pi_{k}\in \left[0,1\right]$ , such that $\sum_{k=1}^{K}\pi_{k}=1$ . The number of mixture components, K , is treated as a hyperparameter and selected via grid search.

The objective is to use this GMM to derive an uncertainty metric, that describes the proximity to OOD measurements during the assembly process. The log-likelihood of an observation, given by

$$L\left(z\right)=\log\left(p\left(z\right)\right),$$ (20)

measures how likely the observation belongs to the GMM distribution. To obtain a normalized uncertainty value, the likelihood is mapped to a logistic score

$$S\left(z\right)=\frac{1}{1+\exp\;\left(\beta \;\left(L\left(z\right)-c\right)\right)}.$$ (21)

The center c and slope β are set by a calibration between the in-distribution (ID) data of D and a synthetic near-OOD dataset $D_{\text{syn}}$ , such that $c=\frac{\tilde{L}\left(D\right)+\tilde{L}\left(D_{\text{syn}}\right)}{2}$ and $\beta =\frac{\log\;\left(\frac{1}{\varepsilon }-1\right)}{\tilde{L}\left(D\right)-c}$ , with $\varepsilon \to 0^{+}$ . The set $D_{\text{syn}}$ is constructed by placing points on a constant squared-Mahalanobis shell around each ID point, where the local geometry is approximated by a responsibility-weighted Gaussian (Battistelli and Chisci, 2014). By tuning the Mahalanobis radius so that the synthetic points lie in the negligible-probability tail of the local distribution, we enforce low uncertainty on ID data and a smooth increase in uncertainty as the system drifts toward unmodeled regimes.

This uncertainty is used to modulate the MPC compliance in real-time, by adapting the stiffness and desired wrench of the CIM via logistic functions. The linear and angular stiffness of the CIM adapt as,

$$K_{i}\left(S\right)=\left(K_{i}^{\;\min}+\frac{K_{i}^{\;\max}-K_{i}^{\;\min}}{1+\exp\;\left(\alpha_{K}\left(c_{K}-S\right)\right)}\right){\hat{n}}_{d,i},$$ (22)

with stiffness bounds $K_{i}^{\;\min},K_{i}^{\;\max}\geq 0$ , and with i denoting the linear or angular part, while the desired contact wrench adapts as

$$\lambda_{d}\left(S\right)=\lambda_{\text{stage}}+\lambda_{d,r},\;\lambda_{d,r}=\left[\begin{array}{c} F_{d,r}\\ r_{EE}\times F_{d,r} \end{array}\right]$$ (23)

$$F_{d,r}=\left(F_{d,r}^{\;\min}+\frac{F_{d,r}^{\;\max}-F_{d,r}^{\;\min}}{1+\exp\;\left(\alpha_{r}\left(c_{r}-S\right)\right)}\right)\;{\hat{n}}_{d}.$$ (24)

here, the parameters α<0,c∈[0,1] denote the slope and midpoint of the logistics; $\lambda_{\text{stage}}$ is the nominal wrench given by the Wrench DMP or the contact exploration, depending on the stage of the assembly; and $F_{d,r}^{\;\min},F_{d,r}^{\;\max}\leq 0$ are the force bounds of the retraction wrench $\lambda_{d,r}$ . The desired bounds and sigmoid parameters are selected empirically to ensure stable and desired compliant behavior during contact. This formulation (Equations 19–24) adapts compliance to the estimated uncertainty: it maximizes compliance when uncertainty is high (e.g., during unfamiliar contacts) by reducing stiffness and the desired contact wrench, and minimizes compliance when uncertainty is low.

## 4 Experimental evaluation

**FIGURE 3:**
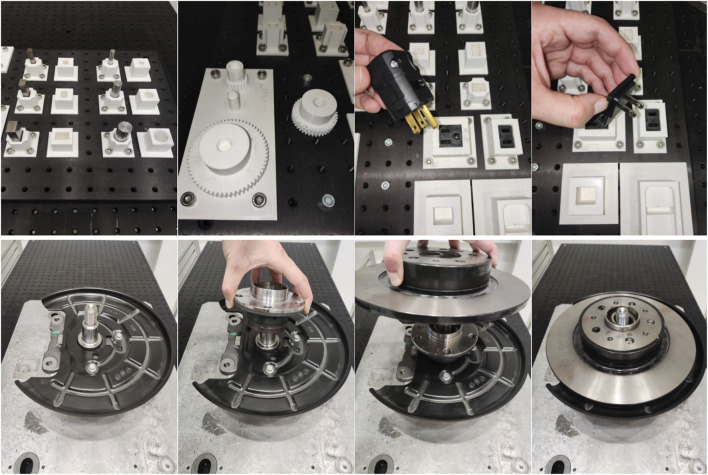
(Top) IndustRealKit parts, (Bottom) Disc brake parts.

In this section, we describe the experimental evaluation of our approach in diverse assembly scenarios, where the assembled parts, as well as their start and goal configuration might change at each trial. The target assembly tasks are defined as:

- IndustRealKit (Tang et al., 2023), Figure 3-top
  - 3x Cylinder Pegs: Small (8 mm), Medium (12 mm), Large (16 mm) must be inserted into corresponding holes (clearances: 0.5–0.6 mm),
  - 3x Orthogonal Pegs: Small (8 mm), Medium (12 mm), Large (16 mm) must be inserted into corresponding holes (clearances: 0.5–0.6 mm),
  - 3x Gears: Small (20 mm), Medium (40 mm), Large (60 mm) must be inserted onto corresponding gearshafts (diametral clearances: 0.5 mm),
  - 2x Plug connectors: i. 2-prong, and ii. 3-prong. Must be inserted into corresponding sockets,
- Automotive (Car) parts (Disc Brake), Figure 3-bottom
  - Wheel Bearing: must be inserted onto the spindle,
  - Wheel Disc: must be placed onto the wheel hub.

We train our framework on three parts only: i. the large cylindical peg, ii. the 3-prong plug, and iii. the wheel bearing. The goal is to generalize to related parts in each case respectively: i. other peg-in-hole variants, ii. the two-prong plug, and iii. the wheel disc. We assume the operator selects the task class (peg-in-hole, plug-insertion, or disc brake assembly) in advance. For each case, the learned, task-specific wDMPs, ART, and GMM models are used.

For each learned assembly, we run multiple trials to reproduce all variations using each of the following controllers:

- CIC: a Cartesian Impedance Controller with joint limits and singularities avoidance utilizing the nullspace, as implemented in Origanti et al. (2025). This controller maintains fixed stiffness which has been determined empirically and does not use force-based contact exploration or alignment detection.
- MPC: standard MPC. This method uses K=0 in Equation 18, and does not use force-based contact exploration or alignment detection.
- MPVIC: MPC with a Cartesian impedance model, which increases stiffness as pose errors grow (inspired by Anand et al. (2023)). This methods sets $\lambda_{d}=\lambda_{\text{stage}}$ and skips the alignment detection phase.
- uMPC-ART: our approach, but without executing a retrial on misalignment.
-
${\text{uMPC}-\text{ART}}_{r^{+}}$ : our approach with retrial.
-
${\text{uMPC}-\text{ART}}_{r^{+}}^{\text{ stiff}}$ : our approach with retrial and with fixed maximum stiffness $K=K_{\max}$ , instead of adaptive via uncertainty.

For all the above, the MPC is solved at 200Hz using the Feasibility-driven Differential Dynamic Programming approach of the Crocoddyl library (Mastalli et al., 2020), which computes robot dynamics via the pinocchio library (Carpentier et al., 2019). The IK solution, computed with the TRAC-IK library (Beeson and Ames, 2015), is configured to find the closest solution to the current state and provides references to the MPC at 200Hz . The KUKA manipulator is equiped either with the Robotiq 2F-85 adaptive gripper (for IndustRealKit parts) or the OnRobot 3FG25 gripper (for car parts). The OnRobot 3FG25 includes an integrated F/T sensor that gives measurements at 140Hz , while the Robotiq 2F-85 setup incorporates an external Robotiq FT300 sensor mounted before the gripper and working at 100Hz .

**FIGURE 4:**
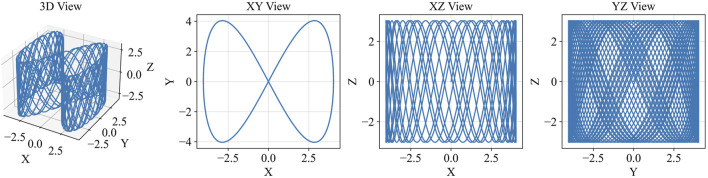
Output of force applied during contact exploration (Equation 10) with: $f_{\text{x}}=2.7Hz$ , $f_{\text{y}}=5.4Hz$ , $f_{\text{z}}=4.5Hz$ .

For contact exploration, we tune the frequency ratios in Equation 10 to symmetrically excite the EE about the estimated goal in the XY plane—defined as the assembly (mating) surface—with Z aligned to the insertion axis (surface normal) (Figure 4).

**FIGURE 5:**
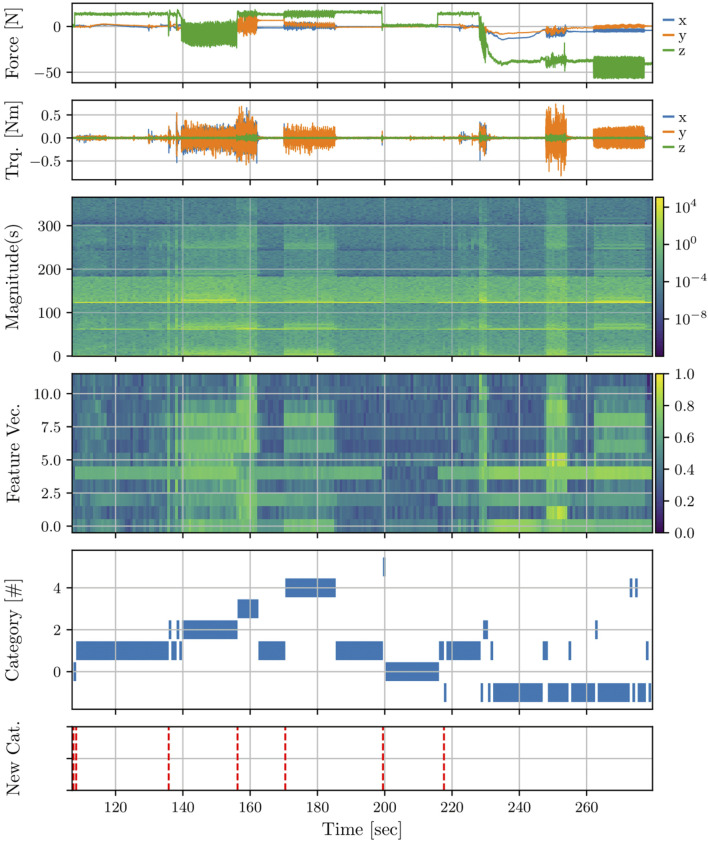
Exemplary data from the ART-based contact classification. From top to bottom: the six channels of the raw measurement (three forces, three torques), the corresponding frequency magnitudes stacked, the magnitudes after max-pooling and scaling, the assigned category index, and indication of novelty (mismatch).

The ART classifier is parameterized by setting the number of samples in a window for the FFT, the overlapping, the size of the blocks used for max pooling, the local and global vigilance parameters, and the scaling to use. When started, it initializes by default with deactivated learning, allowing to pre-train a linear MinMaxScaler of the scikit-learn library (Pedregosa et al., 2011), when the encoded data’s minimum and maximum cannot be estimated in advance. To obtain reasonable values for these parameters, a grid search can be performed on offline data. An exemplary result from such grid search based on a successful and a failed attempt to assemble the wheel bearing is shown in Figure 5. In particular, learning is only activated on the first part of the data (until t=200s ) corresponding to a successful assembly, and tested with the remaining part of the data corresponding to a failed assembly. We observe that in the successful case, the part is initially jammed ( t≈140−155s ) causing high forces in Z-Axis, but the contact exploration resolves the misalignment at about t≈155s . During the alignment check that follows (t≈170−185s) , the classifier learns the corresponding frequency magnitude pattern during correct alignment and maps it to a unique category 4. On the other hand, in the failed attempt to align the parts (t≈265−275s) , neither this pattern nor the pattern of the prior exploration phase (t≈245−255s) is stably recognized due to the dissimilarity to the pattern of correct alignment. This results in a significant number of mismatches, where the current input pattern does not match any of the existing categories. In such cases the classifier returns an identifier of −1 . Further, we use an additional median filter on the category identifier that is output by the classifier to handle spurious occurrences of patterns and instable class assignments. Equivalently, to only capture relevant patterns, the classifier can be operated such that learning is only active during an assembly or alignment check where correct alignment is ensured. In this mode of operation, all other patterns are thus mismatching, which simplifies further processing of the classifiers’ output, as a dedicated labeling of the category identifiers is avoided.

A detailed summary of the selected hyperparameters for DMPs, contact exploration, ART, MPC, GMMs, and the uncertainty settings used in the experimental evaluation is provided in Table 1.

**TABLE 1:** Hyperparameters selection.

Component	Group	Parameter	Value				
DMP	Gains	$\alpha_{\text{x}},\;\beta_{\text{x}}$	25.0, 6.25				
		$\alpha_{\omega },\;\beta_{\omega }$	25.0, 6.25				
		$\alpha_{\lambda },\;\beta_{\lambda }$	25.0, 6.25				
	Basis	N	50				
Dither	Amplitudes	$A_{\text{x}},\;A_{\text{y}},\;A_{\text{z}}$	4.0, 4.0, 3.0				
	Frequencies	$f_{\text{x}},\;f_{\text{y}},\;f_{\text{z}}\;\left(Hz\right)$	2.7, 5.4, 4.5				
	Force bounds	$F_{c,ad}^{\;\min},\;F_{c,ad}^{\;\max}\;\left(N\right)$	−23.5, 0				
	Logistic params	$\alpha_{F},\;c_{F}$	−10, 0.5				
ART	STFT	Window size	120				
		Overlap	0				
	Features	Frequency magnitude range selection	1–61				
		Max pooling	32				
	Vigilance	$\rho_{\text{global}}$	0.93				
		$\rho_{\text{local}}$	0.94				
	Bias	$\alpha_{T}$	0.001				
	Learning rate	b	0.7				
MPC	Stage cost	Q	$blkdiag\left(10^{4}\;I_{n\times n},\;5\;I_{n\times n}\right)$				
		R	$0.01\;I_{n}$				
	Terminal cost	$Q_{T}$	$blkdiag\left(10^{6}\;I_{n\times n},\;500\;I_{n\times n}\right)$				
	Horizon	T (s)	0.45				
GMM	Components	K	Peg	Plug		Car	
			2	3		2	
Uncertainty	Lin. stiffness bounds	$K_{l}^{\;\min},\;K_{l}^{\;\max}\;\left(N/m\right)$	1, 200				
	Ang. stiffness bounds	$K_{a}^{\;\min},\;K_{a}^{\;\min}\;\left(Nm/rad\right)$	0.01, 0.5				
	Logistic params	$\alpha_{K},\;c_{K}$	−10, 0.5				
	Force bounds	$F_{d,r}^{\;\min},\;F_{d,r}^{\;\max}\;\left(N\right)$	−20, 0				
	Logistic params	$\alpha_{r},\;c_{r}$	−10, 0.5				
			Peg		Plug		Car
	Offset	c	−117.5		−110.0		−121.0
	Slope	β	−0.038		−0.038		−0.038

Values used across all experiments.

### 4.1 Results

Tables 2–5 summarize performance across the three task families (peg-in-hole, plug-insertion, and disc brake assembly) under varying start and goal configurations. We report i. the success rate, and ii. the average completion time computed over successful trials. The evaluation comprises three trials for each peg-in-hole instance (three for each cylindrical peg, three for each orthogonal, and three for each gear), five trials per plug type (2- and 3-prong), and five trials per car part (wheel bearing and wheel disc). We discuss the main trends evident in Tables 2, 4 below.

**TABLE 2:** Peg-in-hole: success rates.

Method	Cylinder			Orthogonal			Gears			Subtotal
	L	M	S	L	M	S	L	M	S	Total
CIC	0/3	0/3	0/3	0/3	0/3	0/3	0/3	1/3	2/3	3/27
MPC	0/3	0/3	0/3	0/3	0/3	0/3	0/3	1/3	0/3	1/27
MPVIC	0/3	0/3	0/3	0/3	0/3	0/3	0/3	0/3	2/3	2/27
uMPC-ART	3/3	2/3	2/3	3/3	2/3	0/3	3/3	0/3	3/3	18/27
${\text{uMPC}-\text{ART}}_{r^{+}}$	$3/3^{\#r=0}$	$3/3^{\#r=1}$	$3/3^{\#r=1}$	$3/3^{\#r=0}$	$3/3^{\#r=1}$	$0/3^{\#r=3}$	$3/3^{\#r=1}$	$0/3^{\#r=3}$	$3/3^{\#r=0}$	$21/27^{\#r=10}$
${\text{uMPC}-\text{ART}}_{r^{+}}^{\text{ stiff}}$	$2/3^{\#r=2}$	$3/3^{\#r=0}$	$1/3^{\#r=2}$	$3/3^{\#r=0}$	$3/3^{\#r=1}$	$1/3^{\#r=3}$	$3/3^{\#r=2}$	$0/3^{\#r=3}$	$2/3^{\#r=1}$	$18/27^{\#r=14}$

Each cell shows the number of successful trials over the total trials per part. The superscript #r denotes the number of trials in which a retrial was performed. Abbreviations: L: large, M: medium, S: small.

**TABLE 3:** Peg-in-hole: average completion time [s] of successful trials.

Method	Cylinder			Orthogonal			Gears			Subtotal
	L	M	S	L	M	S	L	M	S	Mean ± std
CIC	–	–	–	–	–	–	–	31.0±0.0 s	30.0±1.4 s	30.3±1.2 s
MPC	–	–	–	–	–	–	–	28.0±0.0 s	–	28.0±0.0 s
MPVIC	–	–	–	–	–	–	–	–	52.5±2.1 s	52.5±2.1 s
uMPC-ART	57.3±1.2 s	61.5±2.1 s	62.0±1.4 s	64.0±1.7 s	62.0±0.0 s	–	59.3±1.2 s	–	62.0±1.0 s	61.1±2.5 s
${\text{uMPC}-\text{ART}}_{r^{+}}$	57.3±1.2 s	81.0±33.8 s	84.3±38.7 s	64.0±1.7 s	89.0±46.8 s	–	79.7±34.1 s	–	62.0±1.0 s	73.9±27.2 s
${\text{uMPC}-\text{ART}}_{r^{+}}^{\text{ stiff}}$	99.0±41.0 s	65.0±1.73 s	128.0±0.0 s	63.3±2.9 s	104.7±35.3 s	119.0±0.0 s	101.3±35.9 s	–	62.0±2.8 s	87.3±30.4 s

“–” indicates no successful trial. Subtotal aggregates across all nine tasks.

**TABLE 4:** Plug insertion and car-parts assembly: success rates.

Method	Plug		Car parts		Subtotal	
	3-prong	2-prong	Wheel bearing	Wheel disc	Plug	Car parts
CIC	4/5	2/5	2/5	3/5	6/10	5/10
MPC	1/5	0/5	2/5	5/5	1/10	7/10
MPVIC	4/5	1/5	4/5	5/5	5/10	9/10
uMPC-ART	5/5	0/5	5/5	2/5	5/10	7/10
${\text{uMPC}-\text{ART}}_{r^{+}}$	$5/5^{\#r=0}$	$3/5^{\#r=5}$	$5/5^{\#r=0}$	$5/5^{\#r=3}$	$8/10^{\#r=5}$	$10/10^{\#r=3}$
${\text{uMPC}-\text{ART}}_{r^{+}}^{\text{ stiff}}$	$4/5^{\#r=3}$	$5/5^{\#r=5}$	$3/5^{\#r=1}$	$5/5^{\#r=1}$	$9/10^{\#r=8}$	$8/10^{\#r=2}$

Each cell shows the number of successful trials over total trials per part. Subtotal columns aggregate the two Plug tasks and the two Car-parts tasks respectively. The superscript #r denotes the number of trials in which a retrial was performed.

**TABLE 5:** Plug insertion and car-parts assembly: average completion time [s] of successful trials.

Method	Plug		Car parts		Subtotal	
	3-prong	2-prong	Wheel bearing	Wheel disc	Plug	Car parts
CIC	33.8±1.0 s	36.5±2.1 s	55.5±4.9 s	40.3±8.5 s	34.7±1.9 s	46.4±10.5 s
MPC	30.0±0.0 s	–	59.5±0.7 s	51.0±0.7 s	30.0±0.0 s	53.4±4.2 s
MPVIC	65.5±3.3 s	64.0±0.0 s	74.3±2.5 s	67.6±0.5 s	65.2±2.9 s	70.6±3.8 s
uMPC-ART	78.6±4.6 s	–	96.4±0.5 s	90.0±0.0 s	78.6±4.6 s	94.6±3.2 s
${\text{uMPC}-\text{ART}}_{r^{+}}$	78.6±4.6 s	151.7±17.6 s	96.4±0.5 s	139.0±44.8 s	106.0±39.1 s	117.7±37.3 s
${\text{uMPC}-\text{ART}}_{r^{+}}^{\text{ stiff}}$	106.8±31.7 s	141.0±5.2 s	108.3±21.6 s	124.8±33.1 s	125.8±26.7 s	118.6±28.8 s

“–” indicates no successful trial. Subtotal columns aggregate the two Plug tasks and the two Car-parts tasks respectively.

Overall, the proposed method markedly outperforms the baselines, exhibiting a clear robustness–speed trade-off. The CIC, MPC and MPVIC baselines rarely succeed on peg-in-hole (CIC: 3/27 ; MPC: 1/27 ; MPVIC: 2/27 ), while on plug and car-parts performance is mixed (respectively, CIC: 6/10 and 5/10; MPC: 1/10 and 7/10; MPVIC: 5/10 and 9/10). In contrast, uMPC-ART generalizes well on all tasks, with 18/27 on pegs, 5/10 on plugs, and 7/10 on car parts. Adding the retrial mechanism, ${\text{uMPC}-\text{ART}}_{r^{+}}$ yields the best reliability overall, achieving 21/27 on pegs, 8/10 on plugs, and 10/10 on car parts. This indicates that the ART-based misalignment detection followed by retrial, significantly improves success in uncertain tight-tolerance insertions tasks. The stiff variant, ${\text{uMPC}-\text{ART}}_{r^{+}}^{\text{ stiff}}$ , further improves plugs to 9/10 but regresses on pegs to 18/27 and on car-parts to 8/10 , suggesting that keeping a fixed stiffness and adapting only the contact wrench via uncertainty is not the best fit for diverse assembly tasks.

**FIGURE 6:**
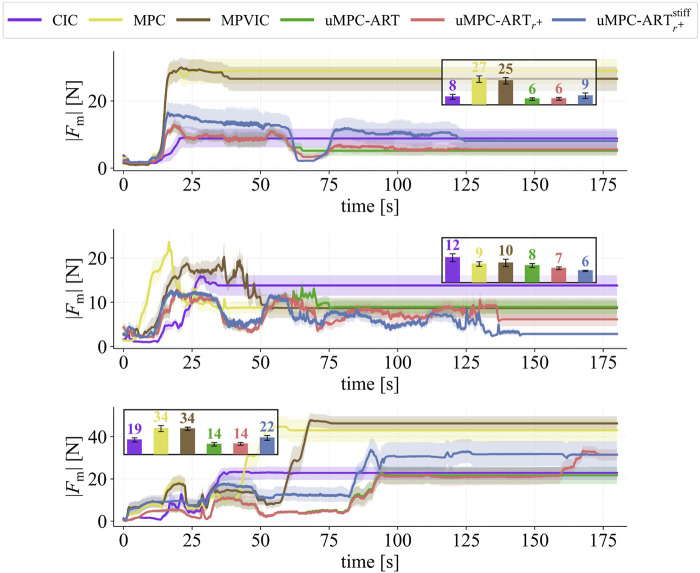
Force measurements over the 47 assembly trials listed in Tables 2–5. (Top) 27x peg-in-hole, (Middle) 10x plug insertion, (Bottom) 10x car-parts. (Inset) Average force for all trials.

**FIGURE 7:**
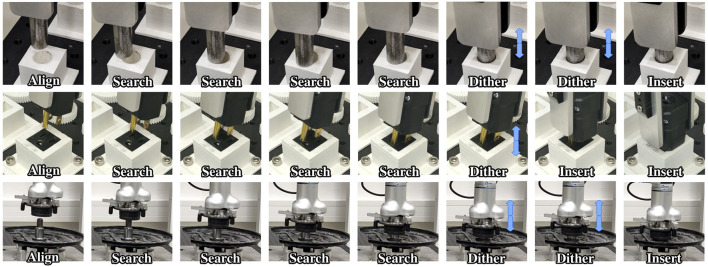
Snapshots of three assembly tasks following our policy. (Top) Medium cylinder peg, (Middle) 3-prong plug, (Bottom) Wheel bearing.

In terms of timing (Tables 3, 5), when the baselines do succeed they are comparatively fast. CIC completes successful trials the quickest on average ∼30−46 s, followed by MPC with ∼28−53 s. MPVIC’s few successful peg-in-hole trials complete around 53s, and on plugs/car parts it averages to ∼65−71 s, which is comparable to uMPC-ART ( ∼61−95 s) but without the same reliability. Adding retrials, ${\text{uMPC}-\text{ART}}_{r^{+}}$ increases these to ∼74−118 s (with Wheel Disc peaking at 139±44.8 s); these longer horizons arise from retrials and the additional contact-exploration steps introduced by our policy. Nevertheless, ${\text{uMPC}-\text{ART}}_{r^{+}}$ improves timing relative to ${\text{uMPC}-\text{ART}}_{r^{+}}^{\text{ stiff}}$ ( ∼ pegs: 87s, plugs: 126s, car-parts: 119s), indicating that adaptive compliance often benefits not only success but also completion time. This is a result of the fact that the stiff approach typically applies higher interaction forces, as seen in Figure 6, which hinders misalignment resolution during contact exploration and thus leading more often to a retrial. In contrast, with adaptive compliance the interaction forces remain among the lowest in most trials and tasks, reducing them by ∼31% vs. CIC, ∼53% vs. MPC, ∼55% vs. MPVIC, and ∼18% vs. its stiff ablation, thereby enabling more efficient search of the contacted surface (Figure 7) and, in turn, more frequent success.

**FIGURE 8:**
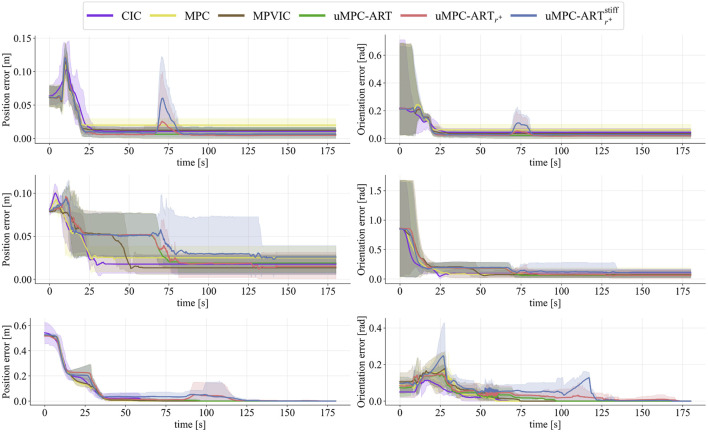
Distance to final position (Left) and orientation (Right) goals. Results over the 47 assembly trials listed in Tables 2–5: (Top) 27x peg-in-hole, (Middle) 10x plug insertion, (Bottom) 10x car-parts.

This trend can also be seen in Figure 8, which plots the distance to the final goal pose over time for all tasks and trials. Independantly of the parts, or start and goal configurations, the ${\text{uMPC}-\text{ART}}_{r^{+}}$ variant (red) maintains the lowest steady-state residuals with tight variability. Although the retrial policy produces brief mid-trajectory spikes, corresponding to the automatic retreat and re-attempt, these are followed by renewed convergence. Notably ${\text{uMPC}-\text{ART}}_{r^{+}}^{\text{ stiff}}$ (blue) triggers retrials more often, as evidenced by larger retreat curves and greater dispersion than ${\text{uMPC}-\text{ART}}_{r^{+}}$ .

Aggregating across all tasks in Table 6, the trend is unambiguous: ${\text{uMPC}-\text{ART}}_{r^{+}}$ achieves the highest overall success (83.0%), followed by the stiff variant, ${\text{uMPC}-\text{ART}}_{r^{+}}^{\text{ stiff}}$ (74.5%), and non-retrial uMPC-ART (63.8%), while MPVIC (34.0%), CIC (29.8%), and MPC (19.1%) trail. Taken together, the results support the following takeaways: i. the classic CIC and MPC methods generalize poorly to different geometries, start and goal configurations of the assemblies, because even millimeter-scale misalignments due to modelling or goal-estimation errors lead to failure when the controller cannot actively explore the contact surface; ii. MPVIC performs better but still degrades under variation, because it increases stiffness during contact, which results in higher interaction forces and thus the parts being stuck because of static friction, thereby limiting effective exploration of the contact surface; iii. a similar effect is observed in our approach with fixed maximum stiffness $\left({\text{uMPC}-\text{ART}}_{r^{+}}^{\text{ stiff}}\right)$ but because the desired wrench is still adapted based on uncertainty the approach succeeds more often, although with frequent retrials; iv. Adapting both the stiffness and desired wrench via uncertainty $\left({\text{uMPC}-\text{ART}}_{r^{+}}\right)$ is key for generalization across shapes and pose variations, because it enables compliant and adaptive contact exploration, which lowers interaction forces while searching for the correct alignment; v. a retrial policy after misalignment detection substantially boosts reliability at the cost of longer executions; and vi. whether the increased execution time is acceptable in industrial settings depends on the application requirements. In many production environments, a failed insertion can incur substantial overhead, e.g., because of damaging the parts, or requiring human intervention to reset the process, thus maximizing reliability can be preferable to minimizing nominal cycle time. For regimes with high part variability, uncertain pose estimates, or tight tolerances, the extra time spent on contact exploration and occasional retrials would often be recovered through reduced downtime. Under such variability, prioritizing success makes ${\text{uMPC}-\text{ART}}_{r^{+}}$ a practical default. By contrast, when cycle time is paramount and parts are well-localized and unambiguous, uMPC-ART without retrial can provide a more favorable speed–reliability trade-off.

**TABLE 6:** Overall success across all tasks.

Method	Overall success
CIC	14/47 (29.8%)
MPC	9/47 (19.1%)
MPVIC	16/47 (34.0%)
uMPC-ART	30/47 (63.8%)
${\text{uMPC}-\text{ART}}_{r^{+}}$	39/47 **(83.0%)**
${\text{uMPC}-\text{ART}}_{r^{+}}^{\text{ stiff}}$	35/47 (74.5%)

The bold value represents the overall best performing method.

## 5 Conclusion and outlook

To summarize, we presented a unified, data-efficient framework for fast reprogramming and adaptive reproduction of contact-rich assembly tasks that combines synchronized wrench–motion DMPs with a GMM-based, uncertainty-aware AMPC, and an ART-based contact classifier. Training requires only two demonstrations: i. kinesthetic teaching of wrench and motion profiles, and ii. an assistive reproduction in which the operator minimally guides the robot through a successful assembly, allowing it to collect nominal contact data; Those are used for training ART to recognize correct alignment and for fitting the GMM to model uncertainty in future, novel assemblies. At run time, the robot adapts the MPC contact model based on the GMM likelihood, which is key for enabling adaptive and compliant exploration of the contact surface using force-based dither motions. In parallel, ART infers the contact context and triggers stage transitions in the assembly process (e.g., triggering a retrial under misalignment).

Trained on a single instance each of peg-in-hole, plug insertion, and wheel-bearing assembly, the system successfully completed 39/47 (≈83%) of mainly novel, previously unseen assemblies with varied geometries and start/goal poses. At the cost of slightly increased completion times, our approach consistently outperformed classic CIC/MPC and an error-driven MPVIC baseline in reliability (success rate), while maintaining the lowest interaction forces overall. These results make a step towards flexible manufacturing lines where production requirements can change frequently.

Although we demonstrate the feasibility of such solution using only an F/T sensor and proprioception, our current system has several limitations that also motivate clear future work directions. First, the higher completion times observed in some cases reflect an intentional trade-off between speed and reliability through compliant exploration and recovery behaviors; future work should aim to retain or improve the achieved success rates while shortening cycle time. Importantly, two concrete routes to achieve this are to make exploration more informed and reduce avoidable retrials. The lack of visual feedback in our framework makes exploration less directed, which may increase the number of retrials; integrating vision (and/or richer tactile sensing) could improve pose initialization, guide contact exploration, and reduce unnecessary retries, thereby directly reducing cycle time. Likewise, performance is currently constrained by gripper capability: limited grasp stability can allow parts to shift within the gripper and exacerbate misalignment, suggesting that improved end-effector design or grasp sensing could reduce failure modes and retrials. Second, while requiring only two demonstrations is comparatively data-efficient, it still imposes manual effort; an important direction is to further automate the process via automatic phase segmentation and self-supervised collection of nominal contact data. Third, the approach relies on multiple empirically tuned hyperparameters, particularly in the uncertainty and adaptation mechanisms; reducing engineering effort through principled self-tuning and online calibration of uncertainty and exploration models is therefore a key avenue for future research. Finally, we considered primarily single-contact insertions; extending the framework to multi-contact and multi-stage assemblies, potentially with regrasping and more complex contact-state transitions, remains an important step toward broader industrial applicability.

## Data Availability

The original contributions presented in the study are included in the article/Supplementary Material, further inquiries can be directed to the corresponding author.
